# Suspicion of Postanesthetic Femoral Paralysis of the Non-Dependent Limb in a Horse

**DOI:** 10.3389/fvets.2018.00012

**Published:** 2018-02-07

**Authors:** Alessandro Mirra, Micaël David Klopfenstein Bregger, Olivier Louis Levionnois

**Affiliations:** ^1^Division of Anesthesiology and Pain Therapy, Department of Clinical Veterinary Medicine, Vetsuisse Faculty, University of Bern, Bern, Switzerland; ^2^Swiss Institute for Equine Medicine, Vetsuisse Faculty, University of Bern, Bern, Switzerland

**Keywords:** femoral paralysis, non-dependent limb, postanesthetic, neuropathy, horse, complication

## Abstract

A 15-year-old Selle Francais gelding was presented to the equine referral hospital for treatment of a left guttural pouch mycosis previously diagnosed. After induction, the horse was shortly hoisted by all four feet, moved on a padded surgical table, and positioned in right lateral recumbency. In order to reduce the risk of bleeding during surgical manipulation of the carotid and maxillary arteries, a mean arterial pressure between 60 and 70 mmHg was targeted. After surgery, the horse was moved in a padded recovery box keeping the same lateral recumbency. Four unsuccessful attempts were performed, with the horse always returning to sternal recumbency keeping the left hind limb up. At the fifth attempt, performed 120 min after the end of the general anesthesia, the horse stood up correctly but moderate ataxia and absence of weight bearing on the left hind limb were shown. Both the stifle and the fetlock joint were held in a flexed position and could not be extended properly in order to set the foot on the ground, resulting in a very short step. The horse was calm, not sweating, and willing to move; the muscles of the affected limb were relaxed, and the limb was neither warm nor painful at palpation. Occasionally, the horse flexed the affected hind limb in an exaggerated motion with marked abduction. No additional laboratory analyses were performed. Due to a strong suspicion of neuropathy, a sling support was initiated and a supportive bandage associated with flunixine administration was performed until resolution of the symptoms. The horse fully recovered after 3 days. This case report does not clarify the pathogenesis of the possible postanesthetic neuropathy accounted on the non-dependent limb, highlighting the need for future research in this field. Non-dependent limb neuropathy should be an expected problem even after having ruled out the most commonly known causes predisposing to postanesthetic lameness.

## Introduction

The present case reports the suspicion of postanesthetic femoral paralysis of the non-dependent limb in a horse, an unexpected occurrence, which may induce serious complications.

A 15-year-old Selle Français gelding, with a weight of 570 kg and a body condition score of 4/5 (1) was presented to the equine referral hospital for treatment of a left guttural pouch mycosis. Owner consent for publication of patient’s data was obtained. The infection did not affect any arterial vessel and no bleeding had been reported by the owner or the private veterinarian. Surgery for arterial coil embolization was scheduled (2). Preanesthetic clinical examination revealed a heart rate (HR) of 28 beats/min, a respiratory rate (RR) of 16 breaths/min, a rectal temperature of 37.3°C, and pink mucous membrane with a capillary refill time of 1.5 s. No abnormalities were detected on thorax auscultation and hematology and biochemistry were unremarkable. The horse had been treated for 2 days with local clotrimazol. Therefore, the patient was classified as an American Society of Anesthesiologist 2 category.

General anesthesia-related risks were considered and particular attention was placed on blood pressure management, with an aim to maintain a mean arterial blood pressure (MAP) between 60 and 70 mmHg in order to reduce the risk of bleeding during surgical manipulation of the carotid and maxillary arteries.

As the animal was calm, a 14-G catheter was placed without sedation in the left jugular vein. Acepromazine (Prequillan, Boehringer Ingelheim GmbH, Switzerland) (0.02 mg kg^−1^) was administered intravenously (IV) 30 min before anesthesia induction. The horse was then moved into a padded induction box where xylazine (Xylasol, Graeub AG, Switzerland) (0.6 mg kg^−1^) and, immediately after, levomethadone (l-Polamivet, MSD Animal Health GmbH, Switzerland) (0.05 mg kg^−1^) were administered IV. Marked sedation was achieved within 10 min. Anesthesia was induced in a swing gate with diazepam (Valium, Roche Pharma, Switzerland) (0.05 mg kg^−1^) IV and ketamine (Ketasol, Graeub AG, Switzerland) (1.5 mg kg^−1^) IV (mixed in the same syringe), and, after flushing the catheter with saline, thiopental (Thiopental Inresa, Ospedalia AG, Switzerland) (1.5 mg kg^−1^) IV. The trachea was intubated with a 28 mm internal diameter endotracheal tube. The horse was shortly hoisted by all four feet and positioned in right lateral recumbency onto a padded surgical table. Both hind limbs were positioned parallel to the ground on a specific padded support, with the coxofemoral joint positioned in its spontaneous natural angle. Anesthesia was maintained with isoflurane in oxygen-enriched medical air [fraction of inspired oxygen (FiO_2_) = 55%] *via* a circle rebreathing system using an equine anesthetic machine (SurgiVet, Smiths medical, USA). Intravenous fluid therapy consisted of lactated Ringer’s solution (5 ml kg^−1^ h^−1^). A constant rate infusion of lidocaine (lidocaine 2%, Streuli Pharma AG, Switzerland) was administered IV (1.8 mg kg^−1^ h^−1^). Physiological variables [HR, RR, hemoglobin saturation by pulsoximeter, end-tidal partial pressure of carbon dioxide (ETCO_2_), and invasive blood pressure] as well as FiO_2_ and end-tidal percentage of isoflurane were monitored continuously using a multiparameter monitor (Datex-Ohmeda S/5, Datex-Ohmeda, Finland) and recorded manually every 5 min. A 20G arterial cannula was placed in the lateral metatarsal artery of the non-dependent limb (left). The transducer was zeroed to atmospheric pressure, positioned at the level of the heart, and a fast flush test confirmed normal damping of the system by visual observation.

Initial systolic (SAP), mean, and diastolic (DAP) arterial pressures measured approximately 30 min after anesthesia induction were 115, 74, and 87 mmHg, respectively. Isoflurane concentration was adjusted and a dobutamine IV infusion was prepared to be administered, if necessary. Although the horse maintained adequate ETCO_2_ in spontaneous ventilation, the breathing pattern was very irregular, and intermittent positive pressure ventilation was initiated 30 min after induction of anesthesia (tidal volume of approximately 6 l, RR of 7 breaths/min, peak inspiratory pressure of 12–15 cmH_2_O). At 1 and 2 h after induction of anesthesia, arterial blood was taken for gas analysis, revealing acceptable values (pH 7.36 and 7.33; partial pressure of carbon dioxide 58 and 60 mmHg, partial pressure of oxygen 134 and 130 mmHg; lactate 1.1 and 1.0 mmol l^−1^). The SAP, MAP, and DAP were maintained during the whole procedure between 82 and 98 mmHg, 62 and 70 mmHg, and 41 and 63 mmHg, respectively, without the assistance of dobutamine. General anesthesia and surgical procedure were uneventful, and lasted 190 and 145 min, respectively. Lidocaine infusion was stopped 30 min before termination of anesthesia.

The horse was again shortly hoisted by all four feet, moved to the padded recovery box, and positioned in right lateral recumbency. Romifidine (Sedivet, Boehringer Ingelheim, Switzerland) (0.02 mg kg^−1^) was administered in the recovery box through the venous catheter and the horse was left to recover unassisted. The horse swallowed 20 min after end of anesthesia (EA) and the trachea was extubated. Xylazine (0.1 mg kg^−1^) was administered IV twice at 10 and 15 min after EA because of marked nystagmus. A first unsuccessful attempt to stand was made 40 min after EA, followed by three further unsuccessful attempts. Each attempt was quiet and no injuries were suspected. The horse always returned to sternal recumbency, with the left leg always remaining the non-dependent one. It then stood up correctly on the fifth attempt, 120 min after EA. A recovery score of 2/5 [from 1 (best recovery possible) to 5 (worst recovery possible)] was attributed [following classification from Ref. (3)]. When standing, the horse showed moderate ataxia and absence of weight bearing on the left hind limb. Both the stifle and the fetlock joint were held in a flexed position and could not be extended properly in order to set the foot on the ground, resulting in a very short step. The horse was calm, willing to move, and not sweating; the muscles of the affected limb were relaxed, and the limb was neither warm nor painful on palpation. Occasionally, the horse flexed the affected hind limb in an exaggerated motion with marked abduction. No additional laboratory analyses were performed. As a result of the strong suspicion of neuropathy, a supportive bandage was applied to the left hind limb and the horse was provided sling support. Additionally, flunixine meglumine (Fluniximin, Graeub AG, Switzerland) (1.1 mg kg^−1^) IV was administered every 12 h, as well as thiamine (1.6 mg kg^−1^) and pyridoxine (0.7 mg kg^−1^) IV every 24 h (Corébral, Vetoquinol AG, Switzerland). As the horse was judged able to stand without distress, the sling support was removed on the same evening. Weight bearing progressively improved on the following day without further complication, and complete resolution of lameness was observed the third postoperative day.

## Background

Mortality associated with equine anesthesia has been reported to occur in 0.8–1.8% of elective surgeries, with values rising to 19.5% in emergency cases despite improvements in anesthetic techniques and physiological monitoring (4). The largest mortality studies conducted to date in horse, the Confidential Enquiry into Perioperative Equine Fatalities 1 and 2 (5), reported that fractures and myopathy were responsible for 32% of postoperative mortality in non-colic surgeries. Myopathy may also occur alongside neuropathy, influencing the outcome and potentially increasing the risk of long bone fractures (4). Despite lack of pathognomonic symptoms, myopathy may be discriminated from neuropathy by presence of muscle stiffness and pain, leading eventually to fasciculation, sweating, and anxiety (6–8). Increased serum muscular enzymes and myoglobin, electromyography, ultrasonography, and nerve/muscular biopsy have been used to distinguish between the two conditions (6, 9–11), although clear differentiation between them can be challenging (8). Postanesthetic myopathy/neuropathy has been mostly reported in the dependent limbs. However, non-dependent forelimb neuropathy (12) and suspicion of bilateral femoral neuropathy have been reported (11). The occurrence of postanesthetic myopathy/neuropathy has been hypothesized to be caused by decreased tissue perfusion and oxygenation (8, 9, 12). The principal causes for their development are arterial hypotension, poor surgical table padding, and inappropriate positioning leading to regional blood flow disturbances, local compression, and mechanical tension on the nerves.

Recommendations have been made to avoid postanesthetic myopathy/neuropathy development (8, 9, 13). The horse must be placed on the operating table in a position that does not put any part of its body under strain; regional venous outflow should be allowed and pressure increase within muscle bellies avoided. Limbs should be allowed to settle naturally and be secured without force. When the horse is lying in lateral recumbency, both non-dependent limbs should be supported parallel to the ground, and the dependent forelimb should be pulled forward to reduce pressure on it from the chest. If access to the medial side of the dependent forelimb is required, the upper forelimb can be flexed. When the horse is lying in dorsal recumbency, care should be taken if leg extension locking the patellae is required and procedures longer than 20 min should be avoided in this position. Padding helps to spread the weight of the horse over a larger area of the body and should be deep enough to prevent the body reaching the table. Finally, a MAP >70 mmHg has been recommended to ensure a good muscular perfusion, decreasing the incidence of postanesthetic myopathy (14). Same recommendations apply to the recovery phase but, for practical reasons, the legs generally lie on the floor without support.

## Discussion

Report of neuropathy of the non-dependent hind limb, like in the present case, has not been reported yet to the authors’ knowledge.

Differentiation between myopathy and neuropathy can be challenging. They share common causes like arterial hypotension, poor padding of the surgical table, and inappropriate patient positioning, potentially leading to decreased tissue perfusion and oxygenation. Moreover, neuropathy and myopathy may occur in parallel. However, myopathy is generally associated with muscle stiffness, pain at palpation, sweating, and anxiety. In the present case, absence of these clinical signs led us to exclude a component of myopathy. Nerve compression and stretching with consequent nerve ischemia and hypoxia were suspected to be the most probable mechanism behind the observed dysfunction.

As the affected limb was the non-dependent one, factors such as the table padding and horse positioning, which were standard in the present case (8), were unlikely to be the causes of the problem. Hoisting by all four limbs could have potentially induced nerve stretching leading to neuropraxia, although to the author’s knowledge, this has never been reported. The coxofemoral joint was positioned in its natural resting position, which should not lead to nerve damage.

Hypotension could have been a predisposing factor for the development of neuropathy. Because the non-dependent limb was elevated over the heart level, a decreased hydrostatic pressure could have led to a reduction of muscle perfusion. However, in the present case, the arterial line was placed in the affected limb, and the MAP was maintained between 62 and 72 mmHg during the whole procedure, the lowest value being recorded during coil embolization. Therefore, intraoperative hypotension was unlikely to be the main cause of the postoperative complication. Since the arterial catheter was placed in the affected limb, good correlation between measured invasive blood pressure values and actual limb blood flow was expected. Dobutamine administration could have helped increasing femoral blood flow and possibly nerves perfusion (15–17). However, in equines, its effect on peripheral perfusion and microcirculation is still not well characterized (17–19).

In the present case, IV lidocaine was administered since the beginning of the surgical procedure. If ischemia was involved in the subsequent nerve dysfunction, its administration may have shortened normalization of the clinical condition; indeed, lidocaine has been shown to reduce reperfusion injury (20, 21).

The risk of developing postanesthetic myopathy/neuropathy increases with anesthesia duration (4). In the present case, the anesthesia lasted 190 min, which could have contributed to the development of neuropathy. The horse required further 120 min to stand up after EA. However, the first attempt was made 40 min after EA, and the underlying femoral paralysis leading to ataxia may have made it difficult for the horse to reach a stable standing position. Development of the neuropathy during recovery seems unlikely since the unsuccessful attempts were all quiet and the horse always returned to sternal recumbency keeping the left hind limb as the non-dependent.

In the present case, the clinical presentation suggested a femoral paralysis, while myopathy was considered rather unlikely because there was lack of pain, sweating, restlessness, muscular stiffness, fasciculation, and anxiety when the horse recovered from anesthesia. However, further analyses could have been helpful in the diagnostic process. Unexpectedly, high plasma creatine kinase values within a few hours after surgery may support a diagnosis of myopathy (8). Nevertheless, the same enzyme may be elevated with other causes of postanesthetic lameness such as peripheral neuropathy, because of secondary muscle damage, or a mixed myopathy–neuropathy (9). Aspartate aminotransferase and myoglobin can also be elevated in cases of myopathy, with the latter leading to dark red or red/brown urine production (9). Hypoechoic areas within the muscle, when observed by ultrasonography, suggest muscle fiber disruption and acute injury (22) but the prognostic value of such observation is uncertain (6). Finally, muscle and nerve biopsy (11) and electromyography (10, 23) may distinguish between myopathy and neuropathy. However, in absence of local muscular symptoms, identifying a correct location to take the biopsy may be challenging. Moreover, changes associated with denervation are usually detectable by electromyography only 4–5 days after denervation (24, 25). Because of the strong suspicion of neuropathy, the fast improvement of the clinical condition and the low reliability of possible further investigations, no supplementary tests were conducted.

Different treatments have been described for postanesthetic myopathy/neuropathy (6, 8, 9, 11, 12), but no clear guidelines have been established so far. In case of neuropathy symptomatic treatment with nonsteroidal anti-inflammatory agents, corticosteroids and dimethyl sulfoxide have been suggested to reduce neural edema. If the horse is able to stand, a supportive bandage and sling support could be applied. If needed, sedation and/or analgesia must be provided. In case a concomitant myopathy cannot be excluded, further treatment can be used. Dantrolene sodium has been described to relax muscles in cases with severe muscle stiffness. Fluid therapy should be initiated to prevent kidney dysfunction because of myoglobinurea. Acepromazine may also improve lamellar blood flow, and anticoagulants (heparin, aspirin) have been suggested to prevent platelet aggregation and subsequent laminitis syndrome. Additionally, physiotherapy, massage, and ultraviolet light can be considered. In the present case, no signs of discomfort were shown by the animal and intense supportive therapy was not required.

## Concluding Remarks

We report a strong suspicion of postanesthetic femoral paralysis of the non-dependent limb. This case report does not clarify the possible pathogenesis of the suspected nondependent limb postanesthetic neuropathy, highlighting the need for future research in this field. Adequate padding, prudent positioning, and good perfusion pressure must be always considered important factors for avoiding postanesthetic myopathy/neuropathy in horses.

## Author Contributions

AM, MK, and OL were involved in the care of the patient and were involved in planning this case report. AM was responsible for the conception of this case report and preparation of the first draft. MK and OL provided feedback and were involved in revising the manuscript. All the authors have read and approved the final version of the manuscript.

## Conflict of Interest Statement

The authors declare that the research was conducted in the absence of any commercial or financial relationships that could be construed as a potential conflict of interest.
